# When Treatment Backfires: A Case Report of Sulfasalazine-Induced Hypersensitivity Syndrome in a Moroccan Patient With Crohn's Disease and Literature Review

**DOI:** 10.7759/cureus.78372

**Published:** 2025-02-02

**Authors:** Imane Zouaki, Fatimaezzahra Lairani, Hala Aouroud, Oussama Nacir, Adil Ait Errami, Sofia Oubaha, Zouhour Samlani, Khadija Krati

**Affiliations:** 1 Department of Gastroenterology and Hepatology, Mohammed VI University Hospital of Marrakech, Marrakech, MAR; 2 B2S Research Laboratory, Faculty of Medicine and Pharmacy, Cadi Ayyad University, Marrakech, MAR

**Keywords:** acute generalized exanthematous pustulosis, case report, dress reaction with eosinophilia and systemic symptoms, morocco, sulfasalazine-induced hypersensitivity syndrome

## Abstract

Sulfasalazine-induced hypersensitivity syndrome (SIHS) is a drug-specific variant of the syndrome known as drug reaction with eosinophilia and systemic symptoms (DRESS). It is a severe and unpredictable hypersensitivity reaction that can present with a spectrum of symptoms, ranging from mild rashes and unexplained fever to life-threatening systemic organ involvement. This diversity of symptoms often results in a diagnostic delay and/or misdiagnosis. Despite the serious nature of SIHS and the increasing number of cases being reported in the last decade, there is still no consensus regarding its management, and current approaches are based on case reports. The rarity, the unpredictability, and the seriousness of this condition make it difficult for proper randomized trials to be conducted. In the lack of such studies, case reports like ours are essential to deepen our understanding of this complex reaction. Here, we report a case of an 18-year-old Moroccan patient who was recently started on sulfasalazine for a newly diagnosed Crohn's disease. Three weeks later, the patient presented with a diffuse urticarial rash with a pustular scalp eruption and a fever. His workup revealed hyperleukocytosis with neutrophilia, eosinophilia, and hepatitis along with electrolytic abnormalities and elevated C-reactive protein without a definitive source of infection. The patient also admitted the use of metronidazole and spiramycin for a dental abscess five days prior to his admission. Thus, the diagnosis of SIHS with an associated acute generalized exanthematous pustulosis secondary to metronidazole and spiramycin was then made, and the patient was administered intravenous corticosteroids and oral antihistamines followed by a weaning oral regimen starting at 50 mg of oral prednisolone. Supportive management included intravenous fluids with electrolytes, topical emollients, and topical corticosteroids. His symptoms and biological parameters improved, and he was discharged after three weeks. At the outpatient follow-up two weeks later, he was in full remission.

## Introduction

Sulfasalazine is a widely used medication in the management of inflammatory bowel disease (IBD) and rheumatoid arthritis, valued for its efficacy in controlling inflammation; however, despite its efficacy, its use is not without risk. One of its serious adverse effects is sulfasalazine-induced hypersensitivity syndrome (SIHS). SIHS is a severe and unpredictable hypersensitivity reaction that can present with a spectrum of symptoms, ranging from mild rashes and unexplained fever to life-threatening systemic organ involvement [1]. This diversity of symptoms often results in a diagnostic delay and/or misdiagnosis. The pathogenesis of SIHS is yet to be fully understood, but it is probably multifactorial including genetic predisposition, certain viral reactivations, and drug metabolism polymorphisms [1,2].

Despite the serious nature of SIHS and the increasing number of cases reported in the last decade, there is no consensus regarding its management with different molecules and regimens described in the literature [1]. Current approaches are largely based on case reports, with variable success rates. The rarity, the unpredictability, and the seriousness of this condition make it difficult for proper randomized trials to be conducted. In the lack of such studies, case reports like ours are essential in understanding the clinical presentation, the progression, and the management challenges of SIHS. In this report, we present the case of an 18-year-old male who developed SIHS three weeks after starting sulfasalazine for Crohn's disease associated with acute generalized exanthematous pustulosis (AGEP). By examining this case, we aim to highlight the diverse manifestations of SIHS and the challenges in distinguishing it from other conditions such as AGEP also with similar presentations.

Many cases of SIHS have been previously reported in the literature, but not that many cases in patients with IBD. Thus, we carried out a literature review on SIHS in IBD patients by searching PubMed/MEDLINE between January 2012 and September 2024. Search terms were a varied combination of "DRESS syndrome/drug rash with eosinophilia and systemic symptoms/Sulfasalazine induced hypersensitivity syndrome" and "IBD/Crohn's disease/Ulcerative colitis". This review was conducted as an effort to summarize the available literature on SIHS in IBD patients and in the hope of detecting any epidemiological, clinical, biological, or therapeutic specificities of this syndrome in these patients. Due to the non-specificity of its symptoms, the diagnosis of drug reaction with eosinophilia and systemic symptom (DRESS) syndrome is based on evaluating its probability, and two scores have been validated for this reason. We have used the "RegiSCAR" scoring system to classify the diagnosis of SIHS in the reviewed reports into four categories: "no", "possible", "probable", or "definite" [3]. As for the diagnosis of the overlapping AGEP, we have used the diagnostic score of the EuroSCAR study [4].

## Case presentation

Here, we report the case of an 18-year-old patient followed for spondyloarthritis two years prior to his admission under symptomatic treatment. During his hospitalization in the rheumatology department, the patient reported recurrent fluid diarrhea without mucus nor blood and without rectal nor König's syndrome with atypical abdominal pain evolving in flare-ups interspersed with remissions for nine months with the last episode dating up to three months ago but was asymptomatic at the time of his admission. An endoscopic workup with biopsies was done revealing an ileocolic Crohn's disease. The patient was then prescribed sulfasalazine at the dose of 3 g daily. Three weeks later, the patient presented to our institution with a three-day history of a diffuse, urticarial rash on his trunk, limbs, face, and scalp. Upon his admission, the patient also reported that he was treated by metronidazole and spiramycin for a tooth abscess five days prior but denied any history of infectious diseases such as tuberculosis and any food or drug allergies.

**Figure 1:**
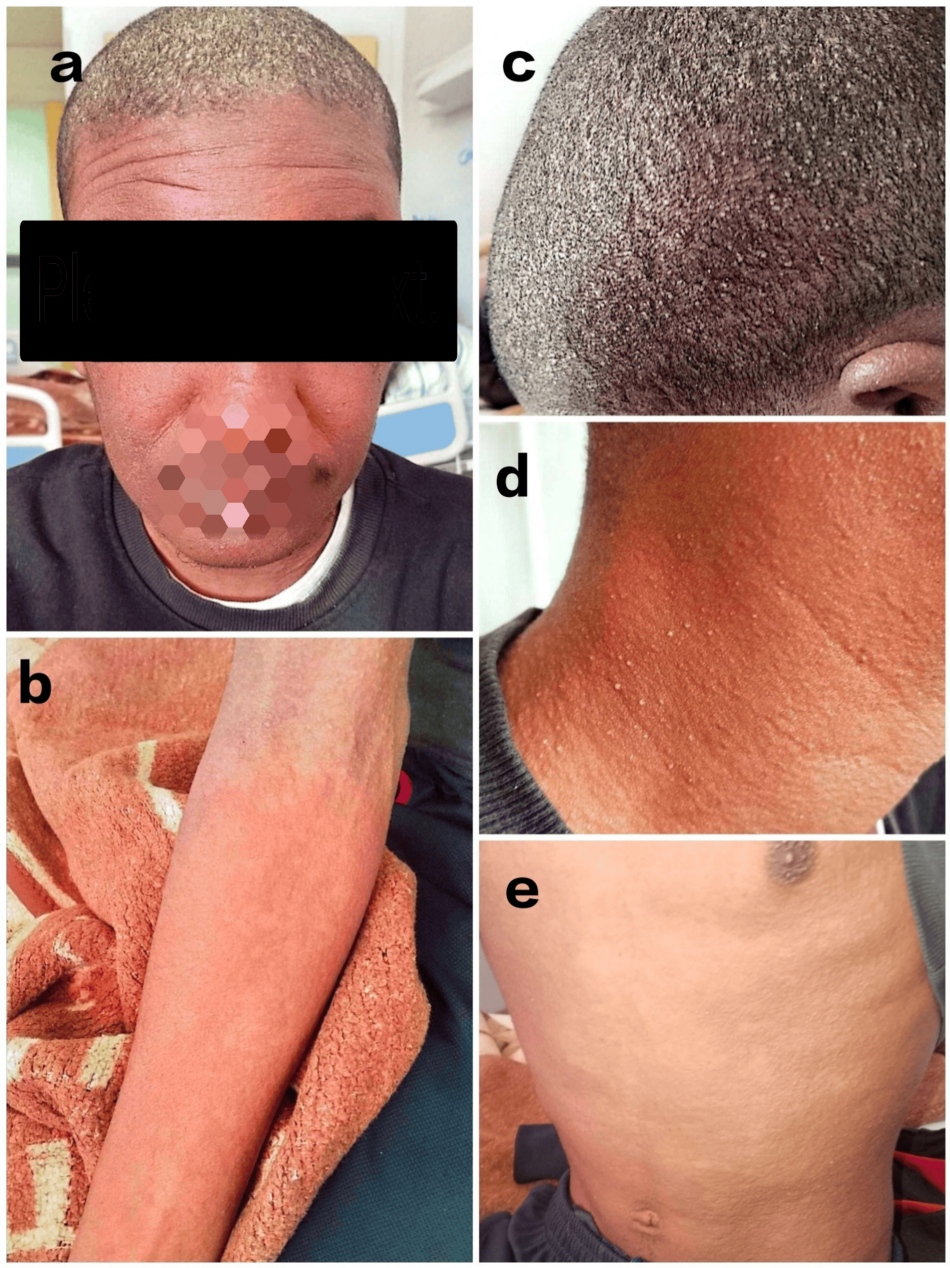
On admission, our patient with diffuse rash over the face and the trunk (a, b, d, e), facial edema (a), and a pustular scalp and neck eruption (a, c, d)

Physical examination revealed a conscious patient, febrile at 38.7°C, tachycardic at 110 beats per minute (bpm), but with a normal blood pressure, a normal respiratory rate at 16 cycles per minute (cpm), and a normal saturation level at 99% on ambient air. We noted scattered urticarial rash on his trunk, limbs, face, and scalp (Figure 1). He also had facial edema, a pustular scalp eruption (non-follicular pustules) (Figure 1), and bilateral cervical and inguinal lymphadenopathy >1 cm. Abdominal and cardiopulmonary examination showed no abnormalities.

His initial workup showed an elevated white blood cell count with eosinophilia, a normal neutrophilic count, and an elevated C-reactive protein (Table 1). His liver workup showed cytolysis with a biological cholestasis (Table 1). Viral hepatitis serologies (VHA, VHB, VHC, EBV, CMV, VIH) came back negative; however, HHV-6 serology was unavailable at our structure. His renal function was normal with a mild hyponatremia and a mild hyperkalemia (Table 1). Pulmonary and urinary tract infections were eliminated. The diagnosis of DRESS syndrome secondary to sulfasalazine was confirmed with a RegiSCAR score of 6 (definite diagnosis). An overlapping AGEP was also confirmed after consulting with the dermatology department secondary to metronidazole and spiramycin with a EuroSCAR score of 8 (definite diagnosis).

**Table 1:** Laboratory findings in our case

Parameters	Patient's values	Reference range
White blood cell count (/uL)	14440	7000-10000
Eosinophilic count (/uL)	3620	<500
Lymphocytic count (/uL)	3300	1000-4000
Neutrophilic count (/uL)	6500	2500-7000
Hemoglobin (g/dl)	9.5	13-18
Alanine aminotransferase (IU/l)	408	10-41
Aspartate aminotransferase (IU/l)	395	10-50
Alkaline phosphatase (IU/l)	291	40-129
Gamma-glutamyl transferase	219	<45
Total bilirubinemia (mg/dl)	9	<10
Creatinine (mg/dl)	5	0-12
Natremia (mmol/l)	129	135-145
Kaliemia (mmol/l)	4.7	3.5-4.5
Lactate dehydrogenase (IU/l)	1147	135-225
C-reactive protein (mg/dl)	70	<5

Sulfasalazine as well as the other treatments were discontinued upon admission, and the patient was administered intravenous methylprednisolone (i.v. mPNL) at 1 mg/kg daily for five days and oral antihistamines. Supportive management included restricted fluid intake for his hyponatremia, topical emollients, and topical steroids. After five days, his symptoms and biological parameters gradually improved. His rash gradually darkened and subsided, and his facial edema, fever, and lymphadenopathy gradually subsided too. He was discharged after a three-week hospitalization on a weaning oral regimen of corticosteroids starting at 1 mg/kg of oral prednisolone.

**Figure 2:**
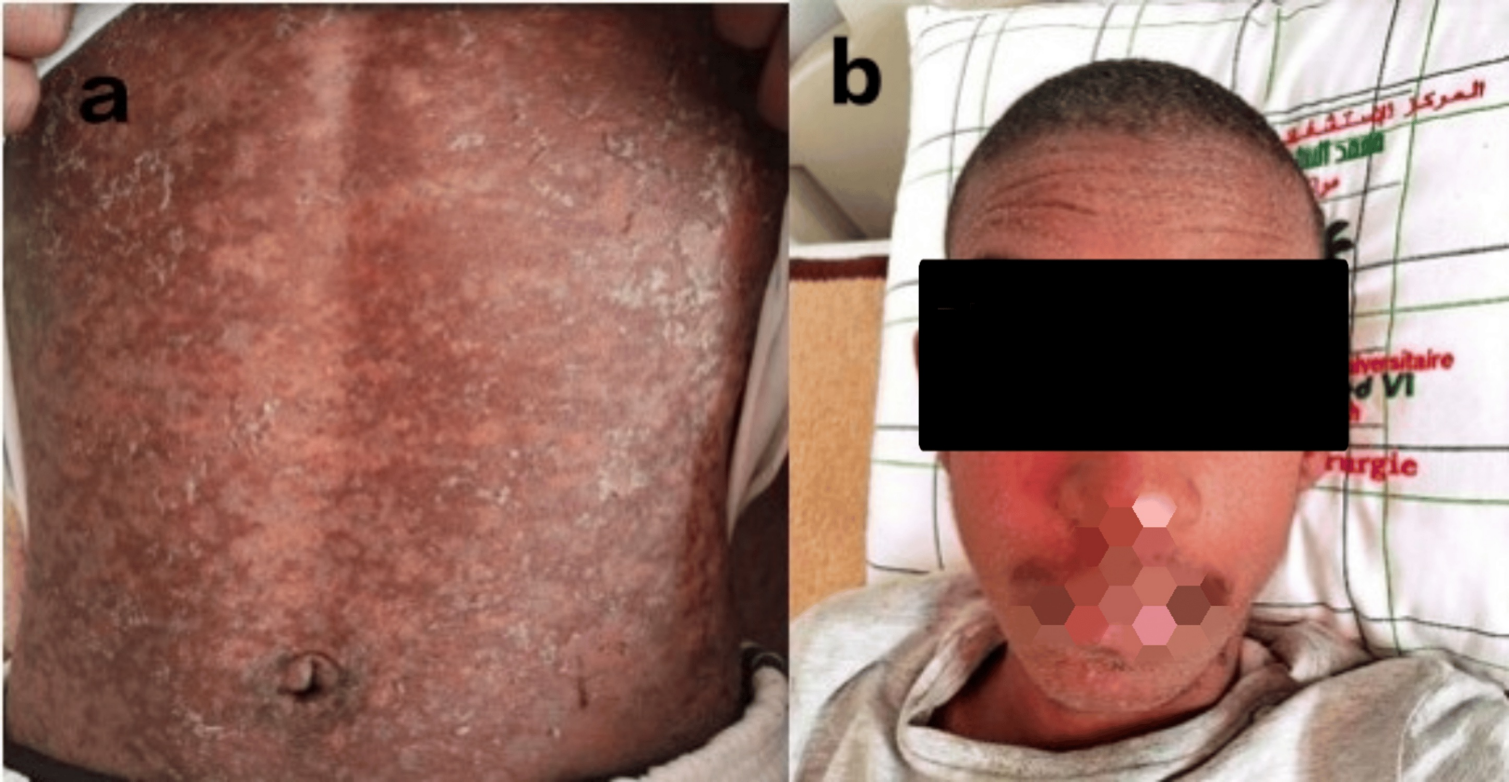
Two weeks post-treatment, the patient shows very subtle post-inflammatory skin changes (a) with regression of facial edema and the pustular eruption (b)

At the outpatient follow-up two weeks later, he was found to be healthy, with very subtle post-inflammatory skin changes and normal liver function tests (Figure 2). As for Crohn's treatment, he is planned to be put on biologics. The incident was reported to the pharmacovigilance department of our hospital.

## Discussion

DRESS syndrome is often linked to anticonvulsants, antibiotics, and sulfonamides, with almost 12% of DRESS cases secondary to sulfonamides and sulfasalazine being accountable for half of them [1]. SIHS is a drug-specific variant of the DRESS syndrome. Due to the wide use of sulfasalazine in the treatment of diverse inflammatory pathologies mainly in rheumatology and in gastroenterology, many cases of SIHS have previously been reported. However, its incidence is not yet defined, and not many cases of IBD patients with SIHS have been reported. Therefore, we have conducted a literature review of cases of SIHS in IBD patients published since January 2012 to see the epidemiological, clinical, biological, and therapeutic characteristics of this population. Table 2 details the major demographic, clinical, and biological findings and treatment characteristics of the reported cases of SIHS in IBD patients.

**Table 2:** Details of SIHS in IBD patients reported in the literature *Year of publication. **Flare-up of the underlying IBD M: male; F: female; CD: Crohn's disease; UC: ulcerative colitis; NM: not mentioned; SSZ: sulfasalazine; IBD: inflammatory bowel disease; ANB: antinuclear antibody; RMSF: Rocky Mountain spotted fever; CRP: C-reactive protein; mPNL: methylprednisolone; RF: rheumatoid factor; GC: glucocorticoids; LMWHs: low-molecular-weight heparin; GCS-F: granulocyte colony-stimulating factor; HLH: hemophagocytic lymphohistiocytosis; LAD: lymphadenopathy; PRED: prednisone; PT: prothrombin time; LDH: lactate dehydrogenase; SIHS: sulfasalazine-induced hypersensitivity syndrome

Study	Year*	Number of cases	Age	Sex	IBD type	Comorbidities	RegiSCAR score	Dose	Onset	Symptoms	Biological findings	Flare-up**	Treatment	Outcome
Kanabaj et al. [5]	2023	1	42	M	Unspecified IBD	Hashimoto disease hip replacement, mother with psoriasis, and daughter with atopic dermatitis	Definite (6)	NM	4 weeks	Fever up to 40°C; diffuse maculopapular rash; pruritus; myalgia; general malaise; pustules on the neck/nose; cervical, axillary, and inguinal LAD; pharyngitis; tonsillitis; and subtle periocular edema	Hepatitis, leucocytosis, eosinophilia lymphocytosis, monocytosis, negative viral serologies. Increased serum level of anti-*Yersinia* IgM antibodies (160 U/ml). Negative tumoral markers, ANB positive with granular fluorescence	NM	i.v. mPNL 1 g daily in the first 3 days, then 32 mg p.o. daily for 7 days, and then tapering dose, antihistamines (i.v.), doxycycline 100 mg twice daily, LMWHs, topical treatment	Good
Chen et al. [6]	2022	1	52	M	UC	-	Probable (5)	-	3 weeks	Fever, rash (face, trunk, limbs), facial edema, pruritus, LAD	Leucocytosis, lymphocytosis, eosinophilia, cytolysis, negative CRP, negative ANB	NM	i.v. mPNL 80 mg, antihistamines, and topical GC, i.v. human immunoglobulin 10 g/d for 2 days and then increased to 25 g/d for 5 days and then only mPNL, gradually reduced to 28 mg orally for 5 days; then discharged on oral mPNL 12 mg	Good
Winward et al. [7]	2021	1	-	F	CD	NM	Possible (3)	-	10 days	Fever, diffuse rash, emesis, hypotension, tachycardia	Pancytopenia, hepatitis, hypocomplementemia, elevated CRP, elevated RF, hyperferritinemia, negative autoimmune and infectious workup	None	SSZ suspension, vasopressors, and conservative measures	Recurrence 10 hours after reintroduction and then no symptom for a year after SSZ suspension
Arce et al. [8]	2020	1	24	F	UC	NM	Possible (3)	-	3 weeks	Fever (38.5°C), rash (trunk, neck, ankles), plantar pruritus, cervical LAD	Eosinophilia, hepatic cytolysis, negative serologies	None	SSZ suspension	Good
Khan et al. [9]	2020	1	27	F	UC	Amoxicillin a week prior	Probable (5)	NM	3 weeks	Fever, rash, congestion, cough, diarrhea, dark stool	Eosinophilia, hepatitis, negative serologies and immunologic panel	Flare-up	i.v. mPNL 1 g daily for 3 days and then oral GC	Good
Ammar et al. [10]	2020	1	45	M	UC	None	Definite (6)	-	45 days	Fever (39.5°C), pruritic rash (face, trunk, and extremities), cervical LAD	Severe liver failure (hepatitis with low PT)	NM	SSZ suspension, GC	Initially good than HHV-6 reactivation and HLH with a fatal evolution
Ahmed et al. [11]	2017	1	21	F	UC	NM	Probable (4)	NM	3 weeks	Fever, diffuse rash, facial edema, sore throat, cervical LAD	Leucocytosis and hepatitis	NM	i.v. mPNL and then oral PRED (dosage not specified)	Good
Sussman et al. [12]	2017	1	16	M	UC	Previously was on mesalamine with good evolution	Probable (4)	NM	3 weeks	Fever, diffuse erythematous rash, abdominal pain, LAD	Hepatitis, leucocytosis, eosinophilia, hyponatremia	NM	i.v. GC, topical GC, and, after 3 days, transitioned from i.v. to oral GC with tapering dose	Good
Fathallah et al. [13]	2015	1	25	M	UC	NM	No case (1)	4 g	8 weeks	Fever (40°C), diffuse rash, facial edema, asthenia, cervical LAD	Leucocytosis and then agranulocytosis, hepatitis, inflammatory syndrome, negative serologies	NM	SSZ suspension+GC at 1 mg/kg/day+G-CSF, prophylactic amikacin (1 g daily), and tazocin (4 g daily)	Good
Hubert et al. [14]	2015	1	62	F	CD	NM	Possible (2)	NM	8 weeks	Chills, fever, diffuse miliary rash, chest pain, cough, dizziness, abdominal pain, depression, edema	Leucocytosis, eosinophilia	NM	SSZ suspension and GC (route, molecule, and dosage NM)	NM
Cherquaoui et al. [15]	2015	1	23	F	CD	None	Definite (7)	-	3 weeks	Fever (38°C), maculopapular generalized rash, asthenia, facial edema, rhinitis, dry cough, purulent conjunctivitis, LADs	Leucocytosis (eosinophilia, basophilia), hepatic cytolysis, negative serologies	NM	SSZ suspension, topical GC, antihistamines, ophthalmic eye drops	Good
Pałgan and Bartuzi [16]	2014	1	54	F	UC	Cholecystectomy	Definite (6)	1 g	20 days	Fever (39°C), diffuse exanthematous rash, tachycardia, cervical and axillary LADs, hepatomegaly	Leucocytosis, eosinophilia, hepatitis, inflammatory syndrome, negative viral serologies	Yes	SSZ suspension, 24 mg/day of dexamethasone which was cut in half after six days	Good
Chebbi et al. [17]	2014	3 (only one with IBD and is considered)	45	M	UC	NM	Definite (7)	-	6 weeks	Fever at 40°C, diffuse maculopapular rash, facial edema, cervical and axillary LADs	Eosinophilia, hepatitis, negative bacterial samples and viral serologies	NM	SSZ suspension, systemic GC therapy at a dose of 1 mg/kg/day	Good
Kang et al. [18]	2012	1	22	M	UC	-	Definite (7)	4.5g	6 weeks	Fever (38.5°C), skin rash, facial edema, splenomegaly	Leucocytosis, lymphocytosis, hepatitis, high LDH	NM	SSZ suspension+i.v. GC (1 mg/kg of PNL equivalent per day) and then oral PNL	Good
Ferrero et al. [19]	2013	1	15	M	Unspecified IBD	NM	Definite (7)	NM	1 week	Fever (up to 40°C), headaches and myalgia, lethargy, diffuse maculopapular rash, facial edema, and cervical, axillary, and inguinal LADs	Leucocytosis, eosinophilia, hepatitis, high CRP, hyponatremia, hypocalcemia, and low magnesium and phosphorus. Bacterial and viral tests for HHV-6 and EBV were negative	Flare-up (bloody diarrhea)	i.v. mPNL 40 mg/12 h and then tapered after 5 days to 30 mg twice daily+doxycycline for potential RMSF+ibuprofen+acetaminophen+diphenhydramine and then a 6-week oral PRED taper starting at 60 mg twice daily	HHV-6 reactivation (acute hepatic failure) and then improvement with GC and discharged on day 16 on a tapering dose of oral GC

On its epidemiology, this syndrome was described in all age groups and in both genders (Table 2). Our patient was an 18-year-old which was within the age range of the published cases ranging from 16 to 62 (Table 2). This syndrome affects both genders with a slight female predominance in the literature [1]; however, in our review, we noted that it affected more male patients with an 8-to-6 male-to-female ratio (Table 2). Our patient was diagnosed with Crohn's disease, while in the literature most cases were of ulcerative colitis (UC) (only three cases of Crohn's versus 10 cases of UC and two of unspecified IBD (Table 2)) likely because sulfasalazine is often prescribed in UC than in Crohn's disease cases as the first line of treatment.

Classically known as the "three-week sulfasalazine syndrome", the onset of symptoms varies from one to six weeks but usually occurs in the three weeks following the start of the treatment as seen in our literature review and in our case (Table 2). The duration between drug exposure and symptoms is delayed when compared to other severe cutaneous adverse reactions (SCARs). The onset is usually marked by flu-like symptoms, fever, myalgia, and pharyngitis [1]. Fever is practically constant in this syndrome, as noted in our case and in all reviewed cases (Table 1). It may precede the cutaneous symptoms by several days or weeks which may further derail the diagnosis towards an underlying infection at first [1]. The cutaneous symptoms are constant and are reported in all cases (Table 1). They are polymorphic but usually manifest with patchy or confluent erythematous macules starting in the face and the upper trunk and expanding downwards [2]. They may include pustular eruption, especially in the neck and/or scalp region, mimicking AGEP as in our case and in the case of Kanabaj et al. [5]. Except in our case, a concurrent AGEP secondary to the intake of metronidazole and spiramycin was in fact confirmed by a definite EuroSCAR score. This simulation can pose a problem of differential diagnosis between the two syndromes; however, AGEP has a usually more acute onset in contrast to the delayed onset of SIHS, and visceral involvement has rarely been described in AGEP [1]. Facial edema is another frequent symptom that was present in our patient and in seven other cases, while generalized edema was reported in one patient (Table 2). The presence of lymphadenopathies, mostly cervical, is also noted in our case and in 11 out of the 14 reported cases (Table 2). Other manifestations included splenomegaly in one case and hepatomegaly in one case (Table 2).

In terms of biological findings, our patient presented with leucocytosis and significant eosinophilia, while neutrophil and lymphocyte counts were within normal limits. Additionally, there was evidence of inflammatory anemia, accompanied by elevated C-reactive protein (CRP) and lactate dehydrogenase (LDH) levels. These findings correspond to the findings of other reported cases also with eosinophilia and an inflammatory syndrome (Table 2) [2]. Atypical lymphocytes are a usual finding and can be found in 27-67% of cases; however, these were not noted in our case [2]. The main internal organ lesion in our case was the liver as was for most reported cases (Table 2) [2]. It manifested with hepatomegaly in the case of Pałgan and Bartuzi and an unexplained hepatitis in most cases (Table 2) [16]. Cardiac involvement was seen in two cases, one with hypotension and tachycardia and the other with an isolated tachycardia (Table 2). Renal or nervous system involvement was not objectified in any of the cases we have reviewed (Table 2). In the recent review of Jasmeen et al. reporting 48 case reports of SIHS, liver involvement was noted in about 77% of the cases followed by the kidney in 35%, then the heart in 19%, and lastly the lungs in 0.02% of the cases [20]. In this review, also no nervous system involvement was noted [20].

Bacterial and immunological workup and viral serologies were negative which further reapplied the diagnosis of SIHS in our case. Such workup was also conducted in most of the reported cases and was negative (Table 1). Unfortunately, we were not able to test HHV-6 due to its unavailability in our structure. However, most consider its reactivation a major criterion to further reapply the diagnosis of SIHS as for the Japanese consensus group [21]. The association of SIHS and the reactivation of HHV-6 are often reported as noted in the cases of Ammar et al. and Ferrero et al. [10,19]. In the review of Jasmeen et al., HHV-6 reactivation was noted in about 23% of the SIHS cases [20]. Unexplained hyponatremia was also noted in our case with a normal renal function as was noted in two other cases of Sussman et al. and Ferrero et al. (Table 2).

The variable clinical presentation makes the diagnosis of SIHS challenging, especially for overlapping SCARs, such as the case of our patient. Thus, some scores/criteria were proposed to evaluate the probability of the diagnosis of each SCAR, and the diagnosis of overlapping SCARs is defined as cases fulfilling the criteria for a definite or probable diagnosis of at least two conditions according to the developed scoring systems [4]. The certainty of an AGEP diagnosis is evaluated using the EuroSCAR score and that of a DRESS syndrome first by the Japanese consensus group Japanese Research Committee on Severe Cutaneous Adverse Reaction (J-SCAR) in 2006 followed by the RegiSCAR group in 2007 [3,21]. The major difference between these two scores is the presence of HHV-6 reactivation for typical DRESS syndrome in the J-SCAR. The RegiSCAR score is most applied especially in North America/Europe and the one we used to evaluate the cases in our report. It is based on the presence/absence of eight variables including fever, eosinophilia, enlarged lymph nodes, atypical lymphocytes, skin involvement, organ involvement, time of resolution, and evaluation of other potential causes with each item scoring from 1 to 2 [3]. Using the RegiSCAR's scoring system, our case was a definite case of DRESS syndrome with seven scoring points. Out of the 14 reviewed IBD cases with SIHS, seven were definite cases, four were probable cases, three were possible cases, and only one was a "no case" (Table 2).

Since the presentation often is not specific to the causative drug, the attribution of DRESS syndrome to a certain molecule is made by evaluating the probability based on factors such as the timing of the drug administration/onset of symptoms, the medication history of the patient, and the known effects of certain drugs.

The basic aspect of DRESS syndrome's treatment is logically the discontinuation of the causative drug, in SIHS cases, sulfasalazine [1,2]. Systematic steroids were used to alleviate SIHS's symptoms in the literature. Their use is justified by their known anti-inflammatory and immunosuppressive effects and is based largely on expert opinion, not on evidence from randomized controlled trials [1,2]. Moreover, different regimens and dosages were described reflecting the lack of standardized guidelines (Table 2). The proposed initial dose is 1 mg/kg/day of prednisone or equivalent followed by tapering doses over a period of at least 6-8 weeks or, in some studies, 3-6 months to prevent relapse or the development of autoimmune responses in the resolution phase [1,2].

In the cases of AGEP that overlap with probable or certain DRESS syndrome, such as ours, it is recommended to treat the patient as a DRESS, with a longer duration of topical or systemic corticosteroids and with progressive tapering over at least three months [4]. In our case, we used pulse i.v. mPNL at the dose of 1 g/kg/day for a period of five days and then switched the patient to an oral weaning regimen of steroids over three months. The corticosteroid regimen was different for each report such as Kanabaj et al. and Khan et al. where a bolus of 1000 mg i.v. mPNL was administered for three days and later switched to oral tapering doses [5,9], while Cherquaoui et al. only used topical steroids and others only oral corticosteroids [15]. Intravenous immunoglobulin can also be used with a usual dose of 0.4 mg/kg but is not recommended in monotherapy. Chen et al. reported their use at the dose of 10 g daily for two days which was then increased to 25 g/d for five days in conjunction with i.v. corticosteroids [6]. In some studies, the use of cyclosporine was also recommended in refractory cases or in case of contradiction of corticosteroids [1,2]. Other treatments include oral or i.v. antihistamines, topical emollients, and/or steroids and in some cases anti-bioprophylaxis with supportive measures [1] (Table 2).

Remission is usually delayed in SIHS. Our patient was in complete recovery in about three weeks from the start of treatment. It was also achieved in most reported cases (Table 2). Unfortunately, this was not the case in the report of Ammar et al. where the evolution was fatal, because of HHV-6 reactivation and hemophagocytic lymphohistiocytosis (HLH) [10]. In their review of SIHS cases, Jasmeen et al. reported a mortality rate of 4.1% (two cases) which was lower than is normally reported for the DRESS syndrome [20]. The mortality rate of DRESS ranges from 1.7% to 8.8% in some studies and 10-20% in others with an increasing mortality rate to as much as 29% in cases of recurrences usually due to hepatic or multiorgan failure [1,2].

The reoccurrence of SIHS upon re-exposure was in fact reported in the literature, and the reaction's onset was fast (10 hours) and more severe such as the case of Winward et al. [7]. That is to underline the importance of educating patients on the indefinite avoidance of not only the culprit drug, in our case, sulfasalazine, but also all drugs that share its chemical structure, i.e., other sulfonamides. The reason is the cross-reactivity between sulfasalazine and other sulfonamide (e.g., sulfamethoxazole) even though they do not share the same indications, one being an anti-inflammatory drug and the other an antibiotic as reported by Zawodniak et al. [22]. This recommendation should expand to involve all first-degree relatives due to the possible genetic predisposition [22].

## Conclusions

Our case report underscores the importance of the early recognition of SIHS in patients presenting with unexplained symptoms after recently starting sulfasalazine not only to prevent the re-exposition to this drug and other sulfonamides but also to administer proper measures promptly and efficiently. It also underlines the possibility of SCARs' overlapping in the same patient if other treatments are being administered. The authors insist also on the importance of declaring such pharmaceutical reactions preventing re-exposure as the recurrence of this condition can augment mortality. Due to the lack of current consensus on the management of SIHS, the authors hope that case reports like ours may provide valuable insights for patients and healthcare professionals, both clinicians and researchers, and help contribute to the understanding of SIHS, ultimately improving patient safety and care. Further studies are much needed to develop specific treatment guidelines.
